# Modulatory Role of Sensory Innervation on Hair Follicle Stem Cell Progeny during Wound Healing of the Rat Skin

**DOI:** 10.1371/journal.pone.0036421

**Published:** 2012-05-04

**Authors:** Eduardo Martínez-Martínez, Claudio I. Galván-Hernández, Brenda Toscano-Márquez, Gabriel Gutiérrez-Ospina

**Affiliations:** 1 Departamento de Biología Celular y Fisiología and Grupo Multidisciplinario de Investigación en Células Troncales IMPULSA 02, Instituto de Investigaciones Biomédicas, Universidad Nacional Autónoma de México, Coyoacán, Distrito Federal, México; 2 Coordinación de Psicofisiología, Facultad de Psicología, Colonia Copilco Universidad, Coyoacán, Distrito Federal, México; University of Pittsburgh, United States of America

## Abstract

**Background:**

The bulge region of the hair follicle contains resident epithelial stem cells (SCs) that are activated and mobilized during hair growth and after epidermal wounding. However, little is known about the signals that modulate these processes. Clinical and experimental observations show that a reduced supply of sensory innervation is associated with delayed wound healing. Since axon terminals of sensory neurons are among the components of the bulge SC niche, we investigated whether these neurons are involved in the activation and mobilization of the hair stem cells during wound healing.

**Methodology/Principal Findings:**

We used neonatal capsaicin treatment to reduce sensory terminals in the rat skin and performed morphometric analyses using design-based stereological methods. Epithelial proliferation was analyzed by quantifying the number of bromodeoxyuridine-labeled (BrdU^+^) nuclei in the epidermis and hair follicles. After wounding, the epidermis of capsaicin-treated rats presented fewer BrdU^+^ nuclei than in control rats. To assess SC progeny migration, we employed a double labeling protocol with iododeoxyuridine and chlorodeoxyuridine (IdU^+^/CldU^+^). The proportion of double-labeled cells was similar in the hair follicles of both groups at 32 h postwounding. IdU^+^/CldU^+^ cell proportion increased in the epidermis of control rats and decreased in treated rats at 61 h postwounding. The epidermal volume immunostained for keratin 6 was greater in treated rats at 61 h. Confocal microscopy analysis revealed that substance P (SP) and calcitonin gene-related peptide (CGRP) receptor immunoreactivity were both present in CD34^+^ and BrdU-retaining cells of the hair follicles.

**Conclusions/Significance:**

Our results suggest that capsaicin denervation impairs SC progeny egress from the hair follicles, a circumstance associated with a greater epidermal activation. Altogether, these phenomena would explain the longer times for healing in denervated skin. Thus, sensory innervation may play a functional role in the modulation of hair SC physiology during wound healing.

## Introduction

Both clinical and experimental observations indicate that sensory neurons are involved in the process of wound repair. A reduction of the cutaneous sensory innervation is generally associated with longer times for healing [1], [2], [3], [4]. These findings imply that the function of dorsal root ganglion (DRG) or sensory neurons is not restricted to receive and transduce mechanical and noxius stimuli (afferent function). Over the past few years, a growing body of evidence documents that sensory nerves regulate physiological and pathological process in the skin and other tissues by activating target cells that express specific receptors for neuromediators (efferent function) [5], [6]. With the recent development of antibodies against sensory neuropeptides and other neuronal markers, it has been possible to establish that sensory nerve terminals occupy very precise positions on skin compartments rather than a random distribution. Both epidermis and hair follicles are predominantly innervated by small sensory neurons with unmyelinated C-fibers and poorly myelinated Aδ-fibers. The nerve terminals of these neurons are characterized for its ability to release a variety of neuromediators which include neuropeptides such as CGRP and SP. Thus, this anatomical relationship raises the question whether nerve terminals from the DRG may be involved in maintaining epithelial tissue homeostasis.

Epithelial homeostasis is a process that depends on the presence of stem cells (SCs). Recent studies have documented that there is a group of SCs residing in the region called the bulge which comprises the lower end of the permanent portion of the hair follicle [7], [8], [9]. These SCs participate in hair growth and epidermal repair. Epidermal wounds result in bulge SC activation and in the mobilization of transient-amplifying cells to the epidermis which ensures the acceleration of reepithelialization [7], [10], [11]. Although in recent years great advances have been made about the intracellular pathways involved in the maintenance of SC quiescence and self-renewal, less is known about the molecular signals coming from the surrounding tissue that influence bulge SCs [12]. These signals may come from cellular elements such as blood vessels, fibroblasts and nerve endings which altogether form the bulge SC niche. In this regard, it has been shown that peripheral neurons are involved in the retention of hematopoietic SCs in the bone endosteal surfaces [13]. As mechanisms and molecular effectors are conserved among SC niches, this finding suggests that the peripheral nervous system may influence the physiology of other SC niches [14]. Nevertheless, it has not been investigated whether sensory derived signals are among the factors that govern SC exit from the bulge and mobilization to the epidermis.

Neonatal capsaicin treatment, which reduces nerve supply to the skin by eliminating small DRG neurons, provokes delayed reepithelialization of full-thickness skin wounds [4], [15]. Noteworthy, the exogenous administration of CGRP and SP promotes wound closure, whereas the lack of CGRP or the administration of a CGRP antagonist results in delayed wound healing [15], [16], [17], [18]. However, the mechanism by which DRG neurons beneficiate wound closure remains poorly understood. Sensory neuropeptides, especially CGRP, can promote epithelial proliferation both in vivo and in vitro [19], [20]. Additionally, the sensory nerve terminals associated with bulge region and perifollicular epidermis undergo fiber remodeling during hair growth cycle and wound healing [21]. Overall, these data raise the possibility that sensory innervation may influence bulge SC and their progeny. In this study, we used neonatal capsaicin treatment to explore whether sensory nerves activate bulge SCs after an epidermal wound and promote cell migration from the follicles to the epidermis to accelerate reepithelialization. By employing design-based stereology analysis, we followed the fate of cutaneous epithelial cells labeled with thymidine analogs under different protocols. Our results indicate that sensory innervation is involved in the activation of epidermal progenitors around the wound, but not follicular progenitors. In addition, sensory nerves facilitate SC bulge progeny traffic to the epidermis. Finally, we find that bulge SC niche contains receptors to neuropeptides which suggest that peptidergic neurons could be involved in the epithelial repair and hair growth.

## Results

### Sensory Denervation Reduced the Activation of Epidermal Cell Proliferation

**Figure 1:**
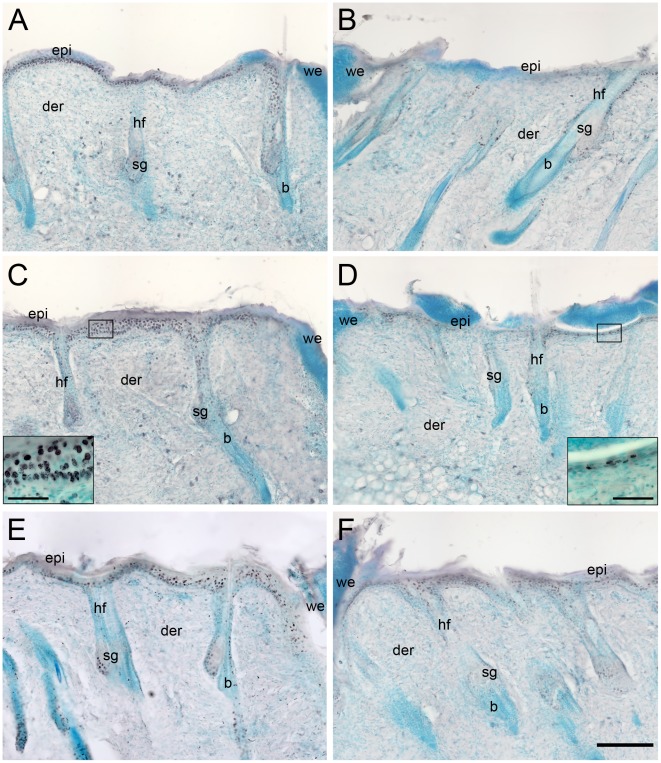
Epidermal thickness was increased after wounding in control rats but not in capsaicin-treated rats. Skin sections from control (A, C, E) and capsaicin-treated rats (B, D, F) at 31 h (A, B), 47 h (C, D), and 61 h (E, F) were inmunostained for BrdU. The epidermis of control rats presented more BrdU^+^ nuclei during the first 47 h postwounding (A–D). Note that the epidermis of treated rats was thinner in the same time window (B, D). The insets in C and D show high magnification of BrdU labeled nuclei in the epidermis. epi, epidermis; der, dermis; sg, sebaceous gland; b, bulge; we, wound edge; hf, hair follicle. Scale bar = 200 µm; inset scale bar = 25 µm.

**Figure 2:**
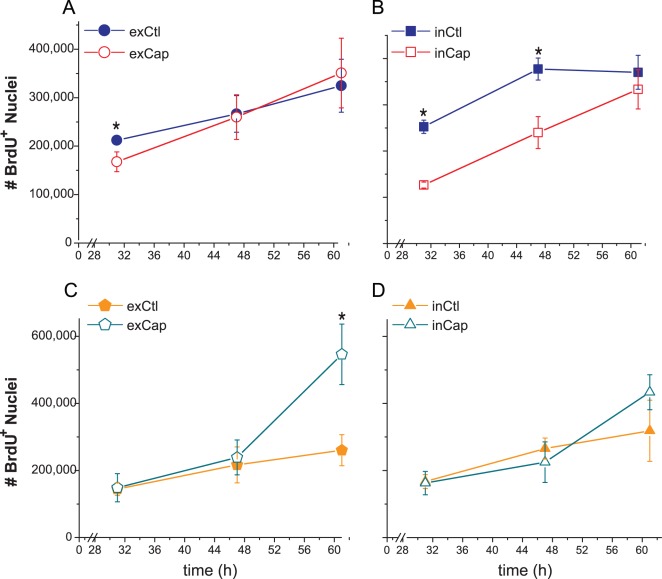
Epidermal proliferation of capsaicin-treated rats was reduced in the proximal region to wound edge. The total number of BrdU^+^ was estimated in the epidermis and hair follicles around the wound with the optical fractionator probe. For quantification we considered two regions: the inner region and the outer region from the wound edge. The total number of BrdU^+^ nuclei for the epidermal outer region is shown in A (exCtl, exCap) and for the inner region in B (inCtl, inCap). C and D show the number BrdU^+^ nuclei in the hair follicles in the outer and inner regions, respectively.

To determine whether chemical denervation altered the cell proliferation of the skin epithelium after wounding, we injected a single pulse of BrdU to control and capsaicin-treated rats. The BrdU labeled (BrdU^+^) nuclei were quantified at 31, 47, and 61 h postwounding. The epidermis proximal to the edge of the wound was thicker in control rats than in treated rats at 31 h and 47 h (Fig. 1). A feature common to both groups was that the number of labeled nuclei decreased with increasing distance from the edge of the wound. However, the labeled nuclei in the case of control rats were more concentrated toward the edge of the wound, whereas the epidermis of the treated rats showed a relatively homogeneous distribution of labeled nuclei throughout the activated epidermis. The BrdU^+^ nuclei were counted in a region spanning 2 mm around the wound (inner region) and 2 mm from the edge of the inner region (outer region). The number of BrdU^+^ nuclei in the epidermis of the inner region was 50% lower in treated rats at 31 h postwounding, 36% lower at 47 h, and with no difference at 61 h (Fig. 2B). In the epidermis of the outer region, the number of BrdU^+^ nuclei was decreased in the treated rats by 21% only at 31 h postwounding (Fig. 2A). In the follicles of the inner region, there were no differences between both groups (Fig. 2D). In contrast, the number of labeled nuclei in the follicles of the outer region was 110% higher in the capsaicin-treated rats than in control rats at 61 h postwounding (Fig. 2C). Interestingly, wounding induced hair growth in some follicles at apparently random spots around the wound.

### Follicle Cell Migration is Impaired in Capsaicin-treated Rats

**Figure 3:**
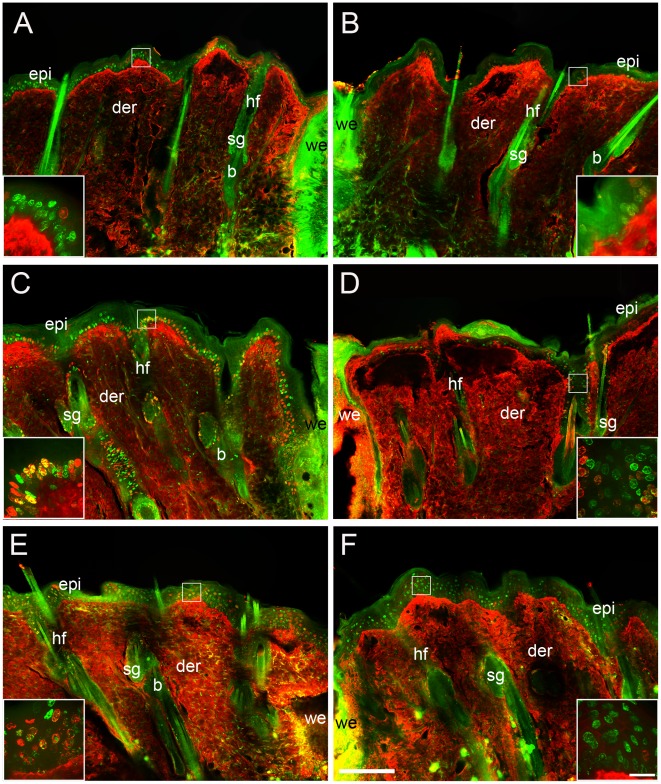
Double-labeled cells increased in the epidermis of control rats. Control (A, C, E) and treated rats (B, D, F) were injected with a single dose of IdU 21 h after wounding. Ten hours later both groups were injected with a dose of CldU and the samples were collected at 32 h (A, B), 41 h (C, D), and 61 h (E, F) after wounding. The label for IdU is shown in green and CldU in red. Note that despite the fact that epidermis of capsaicin-treated rats showed homogeneous distribution of labeled cells (F), they are predominantly IdU^+^ cells. The red staining of the dermis is unspecific binding of the anti-rat antibody. The insets in each panel show high magnification of IdU/CldU labeled nuclei in the epidermis. epi, epidermis; der, dermis; sg, sebaceous gland; b, bulge; we, wound edge; hf, hair follicle. Scale bar = 200 µm; inset scale bar = 25 µm.

To document possible shifts in the flow of keratinocytes from the SC niche of the follicle to the epidermis, we used a double labeling protocol with two thymidine analogs, IdU and CldU. Each of these analogs can be detected independently and are used to label and follow cell populations with different dynamics of proliferation. Therefore, the upper follicular cells can be distinguished because they divide more rapidly than the cells of the epidermis [7], [22]. In the control rats, the double labeled cells (IdU^+^/CldU^+^) were concentrated mainly in the upper portion of the follicle (infundibulum) and in the basal layer of the perifollicular epidermis at 41 h postwounding. At 61 h the IdU^+^/CldU^+^ cells were also present in the suprabasal layers and evenly spread throughout the epidermis (Fig. 3C and 3E). In contrast, capsaicin-treated rats did not present IdU^+^/CldU^+^ cells concentrated in the perifollicular region at any time point. The few double-labeled cells in treated rats were distributed throughout the epidermis (Fig. 3B, 3D, and 3F). Noteworthy, the IdU^+^/CldU^+^ cells were concentrated at the upper part of the follicles in the treated rats at 41 h postwounding (Fig. 3D and Fig. S1).

**Figure 4:**
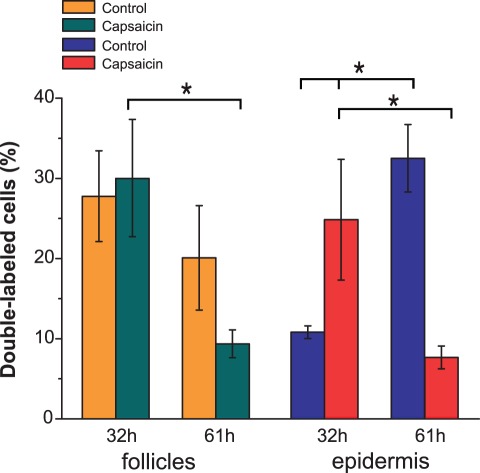
The migration of follicular cells toward the epidermis is altered in capsaicin-treated rats. The percentage of IdU^+^/CldU^+^ in the epidermis and hair follicles was determined at 32 h and 61 h postwounding from the total number of IdU^+^. The proportion of epidermal IdU^+^/CldU^+^ nuclei increased over time in the control group, whereas in the treated group decreased over time.

The migration of the follicular cells was analyzed by calculating the percentage of double labeled cells from the total labeled cells (IdU^+^ cells) in both the hair follicles and the epidermis. In control rats the proportion of IdU^+^/CldU^+^ cells increased in the epidermis from 32 h to 61 h postwounding, while the proportion of these cells in the follicles tended to decrease (Fig. 4). In contrast, the proportion of IdU^+^/CldU^+^ cells in the epidermis of treated rats was higher than in control rats at 32 h and this proportion decreased by 61 h postwounding. In addition, the proportion of double-labeled cells in the hair follicles significantly decreased from 32 h to 61 h in the capsaicin-treated rats.

### Sporadic Events of Apoptosis Occurred in the Hair Follicles of the Capsaicin-treated Rats

**Figure 5:**
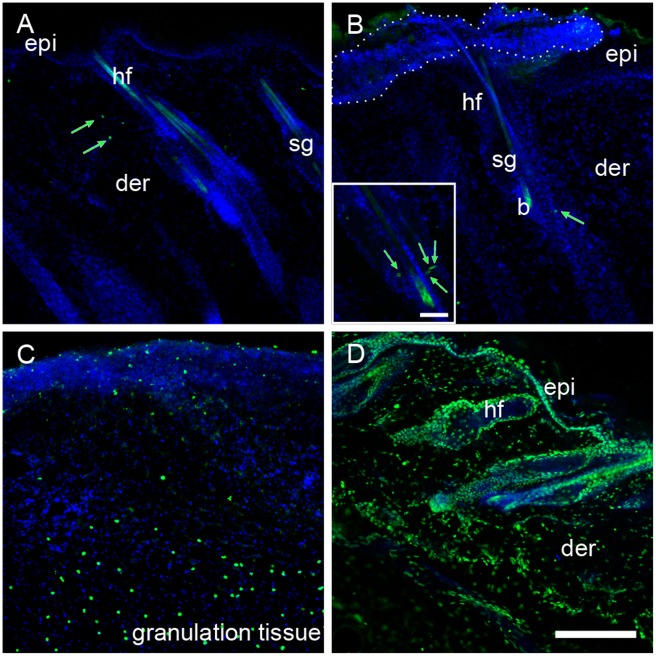
Sporadic cell death was detected in the hair follicles of capsaicin-treated rats. Epithelial apoptosis was evaluated by terminal dUTP nick end-labeling (TUNEL) assay in skin sections of control (A) and capsaicin-treated rats (B) at 51 h postwounding. Few stained nuclei for TUNEL (green arrows) were observed in the hair follicles of treated rats (B). Inset in B shows a higher magnification of a follicle with four labeled nuclei. Note that treated rats (B) presented regions of death epidermis (dotted line). Both groups presented labeled nuclei at the granulation tissue (C). A positive control was prepared by incubating skin sections with DNase I (D). epi, epidermis; der, dermis; sg, sebaceous gland; b, bulge; we, wound edge; hf, hair follicle. Scale bar = 200 µm; inset scale bar = 50 µm.

To examine if the reduced number of IdU^+^/CldU^+^ in the epidermis of treated rats was due to apoptotic cell death, we performed a TUNEL assay and active caspase-3 immunostaining at 51 h and 61 h postwounding. In both groups, the labeling for TUNEL and caspase-3 were observed in the granulation tissue and in some dermal cells between follicles (Fig. 5 and Fig. S2). The capsaicin-treated rats presented only few cells positive for TUNEL (1–4 per follicle) in the upper part of some hair follicles at 51 h (Fig. 5B). Occasionally, the capsaicin-treated rats presented small areas of death epidermis at random positions where it was notorious the presence of a mass of material that lacked organization (Fig. 5B and Fig. S2).

### Epidermal Wound Response is Increased in Capsaicin-treated Rats

**Figure 6:**
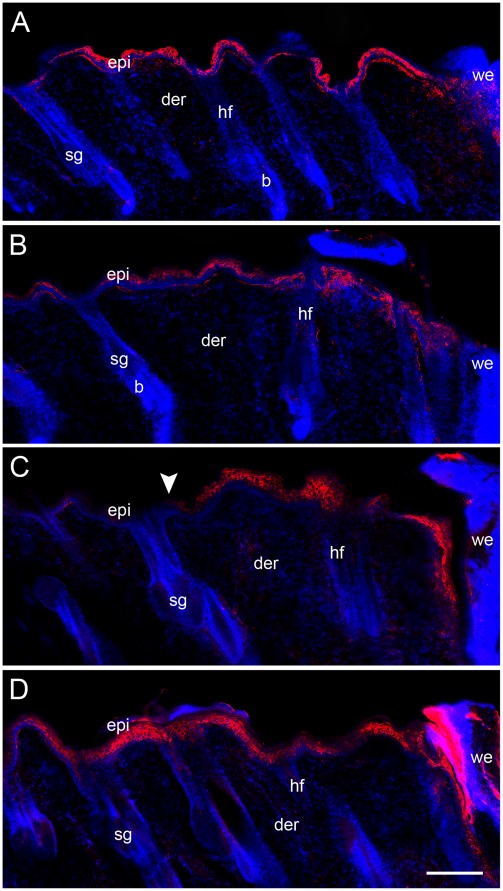
Keratin 6 expression in wounded epidermis. Back skin sections of control (A, C) and treated rats (B, D) were inmunostained for K6, a marker of epidermal activation. The edge of the wound is shown at the right of all the photomicrographs. At 32 h postwounding (A, B), the extension of K6 staining was equivalent in the epidermis of both groups. At 61 h (C, D) we found that the region of activated epidermis is expanded in capsaicin-treated rats when compared with control rats (arrowhead). epi, epidermis; der, dermis; sg, sebaceous gland; b, bulge; we, wound edge; hf, hair follicle. Scale bar = 200 µm.

**Figure 7:**
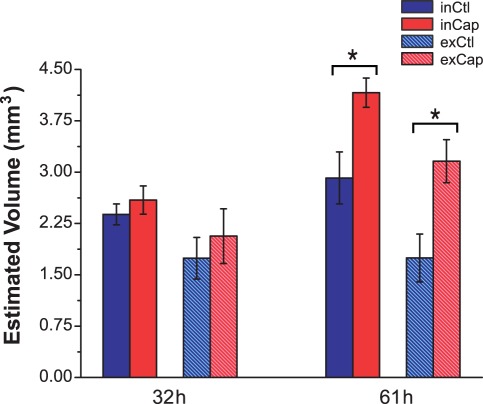
Epidermal activation is extended in capsaicin-treated rats. The graph shows the quantification of the volume of epidermis with K6 expression in control and treated rats. The volume was estimated by the stereological probe of Cavalieri. At 62 h, the volume of distal and proximal regions of epidermis with K6 immunoreactivity was significantly greater in treated rats than in control rats.

To assess whether the epidermis of treated rats turned on some mechanism to compensate for the diminished flow of cells from the follicle, we analyzed the distribution of keratin 6 (K6), a marker of epidermal activation in response to a wound [11]. At 32 h postwounding, the volume of epidermis around the wound labeled for K6 was similar in both groups (Fig. 6A and 6B). However, at 61 h the volume of the epidermis with K6 expression was greater in capsaicin-treated rats (Fig. 6C and 6D). The volume was 43% and 81% higher in the inner and outer region, respectively (Fig. 7). In the treated rats, it was common to observe K6 immunoreactivity beyond the limits of the outer region.

### CGRP^+^ Fibers Decreased in Control Rats After Wounding

The total length of CGRP^+^ fibers was estimated in the epithelium and the dermis to evaluate both the magnitude of sensory denervation after capsaicin treatment and the degree of alteration of the sensory innervation after wounding. In the skin of control rats, the subepdiermal plexus was the origin of the majority of the fibers that penetrate to the epidermis and the fibers that were located around the infundibulum of the hair follicles.

**Figure 8:**
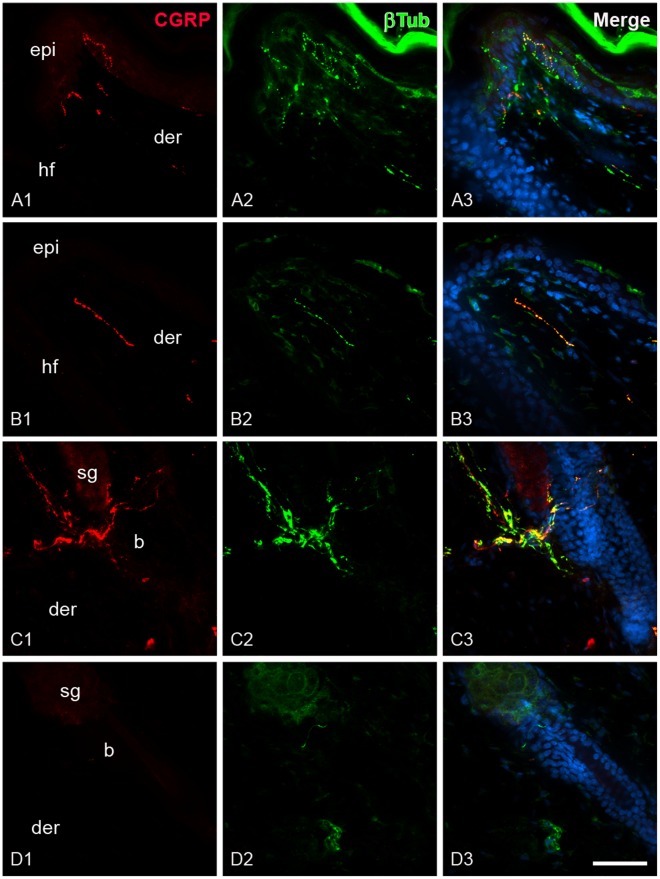
CGRP^+^ and β III tubulin fibers in the epidermis and hair follicles of wounded skin. The panels show photomicrographs of CGRP (A1, B1, E1, F1) and β III tubulin (βTub; A2, B2, E2, F2) immunostaining of skin sections of control (A and E) and treated rats (B and F) at 32 h postwounding. The βTub^+^ fibers were abundantly distributed throughout the epidermis of control rats, while the epidermis of treated rats was practically devoid of this type of fibers. Note that CGRP^+^ fibers in the treated rats were mainly located in the dermis and not associated with epithelium (B). epi, epidermis; der, dermis; sg, sebaceous gland; b, bulge; hf, hair follicle. Scale bar = 50 µm.

Most of the epidermal fibers were labeled for the neuronal marker β-III tubulin, but not for CGRP (βTub^+^/CGRP^−^). The peptidergic (βTub^+^/CGRP^+^) fibers were mainly found at the perifollicular epidermis meandering through keratinocyte layers (Fig. 8A and Fig. S3A). The CGRP^+^ fibers were also surrounding the region below the sebaceous gland (Fig. 8C and Fig. S3C). At 32 h this pattern of innervation was similar in both the outer and the inner region. However, the density of fibers in the epidermis and the hair follicle was diminished in both regions at 61 h postwounding, especially in the inner region. The labeling pattern of the fibers appeared discontinuous at the inner region which was the region where less intraepidermal fibers were observed at this time (Fig. S3E). Also at 61 h, the follicles of the inner region had fewer rings of CGRP^+^ fibers around the bulge area (Fig. S3F).

In the capsaicin-treated rats, the subepidermal plexus was practically nonexistent in the treated rats (Fig. 8B). The intraepidermal βTub^+^/CGRP^−^ and βTub^+^/CGRP^+^ fibers were rare and usually appeared individually and with simple morphology. Also in this group, there were parallel CGRP^+^ fibers to the hair follicle that rarely reached the epidermis (Fig. 8B and Fig. S3G). This type of fibers was not common in control rats. The innervation below the sebaceous gland was present in only some follicles. In those follicles where it was present there were less rings of either βTub^+^/CGRP^−^ or βTub^+^/CGRP^+^ fibers than in controls (Fig. 8D and Fig. S3D and S3H). The innervation pattern in capsaicin-treated rats was similar at 32 h and 61 h postwounding.

**Figure 9:**
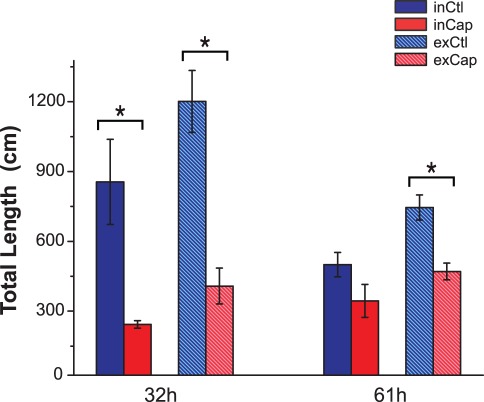
The peptidergic fibers are more abundant in control rats during wound healing. The total length of nerve fibers immunoreactive for CGRP was estimated with the stereological probe of Space balls.

In control rats, the length of CGRP^+^ fibers decreased 43% from 32 h to 61 h in the inner region and 39% in the outer region (Fig. 9). Although the length of CGRP^+^ fibers did not decrease in the treated rats from 32 h to 61 h, the total length of the peptidergic fibers was less than in control rats independently of time point. At 32 h the total length of CGRP^+^ fibers in the treated rats was 73% and 67% lower in the inner and outer region, respectively. At 61 h, the length of CGRP^+^ fibers in the outer region was 38% lower in capsaicin-treated rats than in control rats.

Finally, we also performed a qualitative analysis of SP^+^ fibers which revealed that such fibers are almost absent from the upper dermis of the capsaicin-treated rats, resulting in very few interactions of SP^+^ fibers with the skin epithelium. In contrast, control rats presented SP^+^ fibers at the epidermis, subepidermal plexus and in the nearby of hair follicles (Fig. S4).

### Bulge Stem Cell Niche Express Receptors for Sensory Neuropeptides

**Figure 10:**
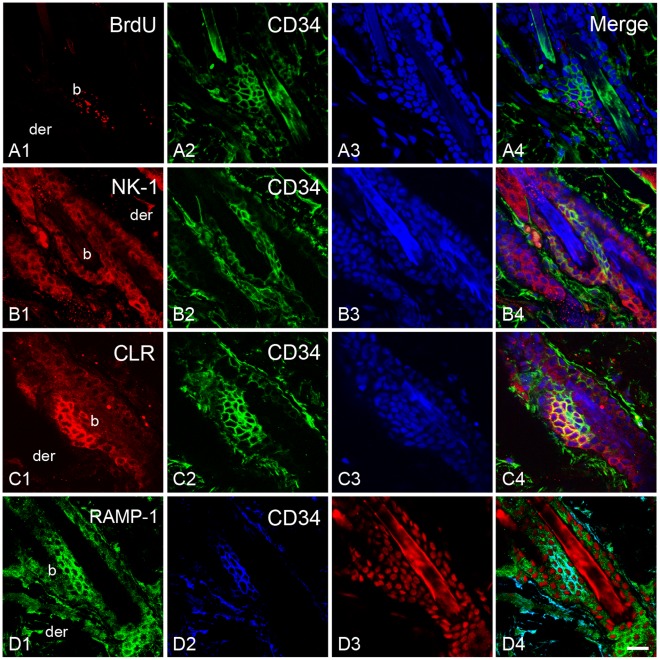
Bulge region of the rat hair follicle express neuropeptide receptors. Similar to mouse skin, label-retaining cells were found in the bulge region of rat hair follicles after 8 weeks of BrdU pulses. By confocal microscopy, we found that bulge region also display CD34 expression (A). Immunoreactivity for SP receptor (NK-1, **B**) and CGRP receptor (CLR, **C**) colocalized with CD34^+^ cells. Note that CLR immunoreactivity was especially intense in the region where CD34^+^ cells were located. The accessory protein RAMP-1, which determines CGRP binding, also colocalized with CD34 expression. Counterstaining was performed with propidium iodide (A3) and TOTO-3 (B3, C3, D3). Scale bar = 20 µm.

**Figure 11:**
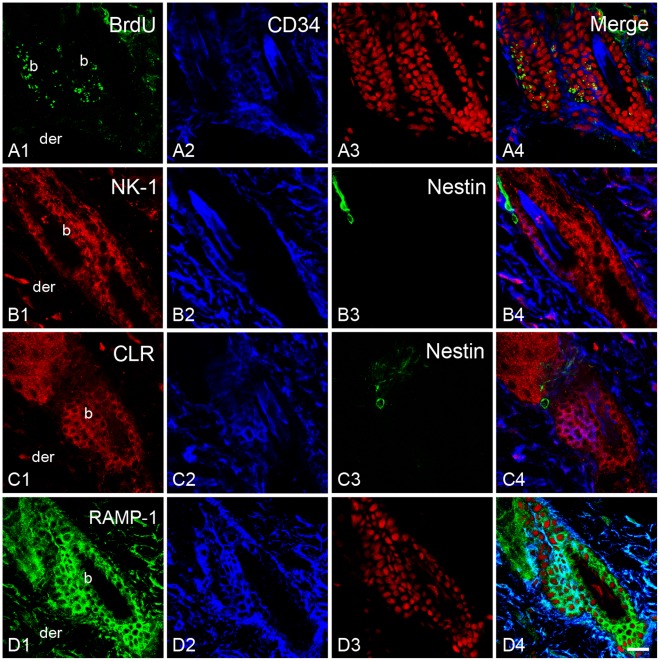
The bulge stem cell niche seems not to be perturbed by neonatal capsaicin treatment. In treated rats, we observed the presence of label-retaining cells and CD34^+^ cell in the bulge region (A). Moreover, NK-1 and CLR colocalized with these markers. Note that dendritic cells immunorreactive for nestin are outside of the hair epithelium and did not colocalize with neuropeptide receptors (B3 and C3). Counterstaining was performed with propidium iodide (A3) and TOTO-3 (C3). Scale Bar = 20 µm.

To evaluate if hair SCs may receive messages from sensory innervation, the presence of neuropeptide receptors in bulge niche was studied by confocal microscopy. In order to locate the SC niche in the rat hair follicle, we employed the bulge marker CD34 and a label-retaining assay. We found that the immunoreactivity for neurokinin-1 (NK-1), SP receptor, was localized in the epidermis and the outer root sheath of the follicle. Notably, the immunoreactivity was less intense in the follicular region. In the bulge region, the label-retaining cells and CD34^+^ cells showed immunoreactivity for NK-1 (Fig. 10B and Fig. S5A). To a similar extent, the calcitonin receptor-like receptor (CLR) immunoreactivity colocalized with the BrdU and CD34 immunoreactivity. However, it was noteworthy that some cells from the bulge region displayed intense immunoreactivity for CLR in comparison with other cells of the outer root sheath (Fig. 10C and Fig. S5B). To determine whether the CLR present in the hair follicle has the ability for binding CGRP, we performed immunostaining for the receptor activity-modifying protein-1 (RAMP-1). The label for this protein was also observed in the outer root sheath. In the bulge region, the label for RAMP-1 colocalized with the label for CD34 (Fig. 10C). The staining pattern of the neuropeptide receptors was similar in capsaicin-treated rats (Fig. 11). In a complementary set of experiments we explored whether a nestin-expressing cell population also displayed the neuropeptide receptors. These cells are pluripotent stem cells that have been implicated in the hair growth cycle [23], [24]. The antibody for nestin labeled a group of dendritic cells that were external to the epithelium of the hair follicle and below the sebaceous gland. In both groups, immunoreactive cells for nestin did not display immunoreactivity for neuropeptide receptors (Fig. 11B, 11C and Fig. S5C and S5D).

## Discussion

Claims that sensory innervation exerts beneficial actions during skin wound repair have been made for decades. Nevertheless, almost all the efforts have been limited to document the impact of denervation upon the time of wound closure [2], [3], [25], [26], [27]. As far as we know, it has not been investigated whether the DRG neurons have a role in the activation or modulation of the epithelial progenitors that participate in reepithelialization. In this work, we found that reduced cutaneous innervation induced by capsaicin impairs bulge stem cell progeny migration to the epidermis during wound healing. We also provided evidence suggesting that sensory innervation differentially modulates SC proliferation of skin epithelia. Overall, we propose that signals derived from DRG neurons are a critical component of the signaling microenvironment that modulates epithelial homeostasis in rodent hairy skin.

In clinical and experimental cases of cutaneous denervation, it has been argued that the delay in wound closure is an unspecific consequence of the recurrent damage of the wound site because of the lack of sensation. However, the fact that capsaicin-treated rodents develop cornea and skin lesions even when self-grooming and scratching behaviors are prevented suggests other possibilities [3], [28], [29], [30]. Recently, we proposed that in conditions of high cell demand and partial denervation some epithelia are not able to generate the number of cells that are required for epithelial expansion [31].The results of the present work point in that direction revealing that sensory nerves are involved in the beginning of the process of reepithelialization. Accordingly, the epidermal proliferation around the excisional wound was impaired during the first 47 h postwounding in the capsaicin-treated rats. In contrast, the initial number of BrdU^+^ cells in the hair follicles was almost identical in both groups. These results may indicate that sensory innervation differentially interacts with distinct populations of epithelial progenitors. Although the low number of BrdU^+^ cells in the epidermis of treated rats during the first 47 h postwounding could result from a reduced number of epithelial progenitors in proliferative phase, this phenomenon could also be a consequence of a reduced mobilization of transit amplifying cells from the follicle. In agreement with this notion, the percentage of IdU^+^/CldU^+^ cells in the epidermis increased over time only in the control group. This effect was not a consequence of a minor activation of progenitors in the hair follicle because at 32 h postwounding there was an equal proportion of double-labeled cells in the follicles of both groups. Apparently, these cells continue with cell division because we observed that IdU^+^/CldU^+^ cell reached a peak in treated rats at 42 h (Fig. S1A). Moreover, the percentage of double-labeled cells in the hair follicle decreases in the treated rats by 61 h, but this reduction was not reflected in an increase of the proportion of IdU^+^/CldU^+^ cells in the epidermis. An additional point worthy of mention is that the fate of the majority of the double labeled cells in the hair follicles of treated rats is not clear. A possible explanation is that the survival of transit amplifying cells depends on sensory fibers. Although, we did not find massive events of cell death, it seems that apoptotic death might be one of the events that impair cell trafficking. Interestingly, it has been shown that SP promotes the survival of human fibroblasts by promoting the expression of BCL-2 which is an inhibitor of apoptosis [32]. An additional function of sensory nerve fibers could be related to control cell cycle duration of some epithelial populations. In support of this notion is the fact that the total number of IdU^+^/CldU^+^ cells in the epidermis of treated rats is relatively constant (Fig. S1B), while its proportion decreases from 32 h to 61 h. Overall these results suggest that deficiencies in the nerve supply hampered the trafficking of follicle cells toward the epidermis. Noteworthy, epithelial cell migration is indirectly promoted by CGRP and SP in cultures of intestinal epithelial cell lines [33], [34]. Although it is arguable that neonatal capsaicin treatment may directly alter keratinocyte functioning, it is important to note that TRPV1 receptor is not functional in keratinocytes probably due to the presence of a dominant negative splice variant (TRPV1b) [35], [36]. Moreover, a TRPV-independent cell necrotic pathway is only induced by high doses of vanilloids [35]. Thus, it is conceivable that if any effect might induce the single dose of capsaicin employed in this study, the affected keratinocytes would be readily eliminated.

Recently, a transgenic mouse that does not have hair follicles in the skin of the tail was used to investigate the functional role of the cells derived from the follicle during epidermal repair after wounding and during postnatal development [11], [22]. From these studies, it was concluded that the cells from the hair follicles are a source of epithelial precursors with high proliferative capacity that allow a rapid epidermal expansion. Although cells from the follicle are not indispensable for the reepithelialization, their presence accelerates this process [11]. This data is in agreement with our observation that capsaicin-treated rats showed a late increase of epidermal proliferation which also reached the level of proliferation found in control rats. Altogether, it seems that in the absence of the arrival of follicular cells, there is an extended recruitment of epithelial precursors, by unknown mechanisms, in the epidermis around the wound. Consistent with this notion, the capsaicin-treated rats presented an increased area of K6 immunoreactivity in the epidermis. The same phenomenon was observed in the hairless tail of the mutant mouse in which the expression of K6 is also extended around the wound [11]. This mechanism of wound repair implies that epithelial precursors migrate for a longer distance to reach the border of the wound which would explain the delay in reepithelialization in denervated tissue. It is also interesting that there was a late increase of cell proliferation in distal follicles from the lesion site in the capsaicin-treated rats. Further studies will be needed to understand the mechanisms that skin employs to promote the activation of distinct regions of the epithelium to better contend with chronic wounds.

A relevant aspect of our findings is that they support the notion that DRG neurons have an efferent role during wound healing. The deficit in epidermal activation and in follicular cell migration could be the result of an insufficient communication between nerve terminals and skin epithelium. The capacity of DRG neurons to regulate biological process is not limited to skin. These neurons have been implicated in a variety of processes such as blood flow and pressure regulation, bone metabolism, neurogenic inflammation, gastric mucosal maintenance, cervix ripening, testicular descent, and tumorigenesis [33], [37], [38], [39], [40], [41], [42], [43]. Consistently, the kind of nerve fibers associated with these processes comes from small DRG neurons which traditionally are only conceived as receptors of noxious information. However, it seems that these neurons are continually and principally serving an efferent effector role via the nonsynaptic terminal release of neuromediators [5]. Thus, the term “noceffector” was coined to recognize the dual role of these neurons [5]. Despite the importance of noceffectors to understand brain and body interactions, there are still several unresolved issues regarding the type of signaling molecules that are involved in this type of communication. Our analysis of sensory innervation revealed that capsaicin treatment eliminated both peptidergic (βTub^+^/CGRP^+^) and nonpeptidergic (βTub^+^/CGRP^−^) fibers in the skin. Therefore, it is likely that substances released from nonpeptidergic terminals also mediate nerve-eptihelial interactions. In fact, nonpeptidergic fibers have been described as the predominant type of fibers in several epithelial tissues of the rat, making them a putative source of signals to keratinocytes that need to be identified (see below) [7], [31]. Another intriguing question about the role of noceffectors during wound healing is whether a similar mechanism operates in glabrous skin which completely lacks hair follicles. Likewise, the human skin is characterized for presenting a very low density of hair follicles. In both cases, it has not been described if there is an analogous reservoir of stem cells as in the rodent bulge region that accelerates the reepithelialization process. In case this reservoir exists, it will be necessary to determine whether nerve terminals are part of the stem cell niche.

The local administration of neuropeptides such as CGRP and SP accelerates wound closure [16], [17]. Although these neuropeptides could influence a variety of processes like the angiogenesis during wound healing [15], our results suggest that sensory innervation influence SC progeny of the rodent hair follicle. Thus, we evaluated whether the SC niche of the bulge has the capacity to receive messages from the nerves. By confocal microscopy, we found that NK-1 and CLR receptors were present in CD34^+^ cells and in labeling-retaining cells from the bulge. This observation is in accordance with previous studies in which it was described that CLR is present in the outer root sheath of human hair follicle [44], [45], [46]. It is possible that CLR protein in the bulge could bind CGRP because RAMP-1 protein is also contained in CD34^+^ cells of the bulge [47]. We note that CLR and RAMP-1 belong to a group of genes that are overexpressed in the bulge region [48]. The latter also seem to occur at the protein level because we observed an intense immunoreactivity of CLR in some cells of the bulge region. A caveat with the use of CD34 as a marker of hair SCs is that it does not label the different stem cell populations that have been recently identified in the isthmus of the hair follicle [49], [50]. However, the fact that neuropeptide receptor immunoreactivity was also observed along the outer root sheath suggests that noceffector neurons may influence other epithelial stem cell populations in the hair follicle. In regard to other skin stem cells, it has been documented that nestin-expressing cells are a population of pluripotent stem cells that reside below the sebaceous gland throughout the hair cycle [23], [24]. The precise role of nestin-expressing cells in hair biology has not been defined. We observed that immunoreactive cells for nestin are external to the hair follicle and did not show immunoreactivity for neuropeptide receptors. Based on its location around hair follicle, it is difficult to consider that nestin-expressing cells have a direct role in the reepithelialization process. However, it is noteworthy that the cellular processes that emerge from its soma were in intimate contact with nerves (data not shown), suggesting some kind of functional interaction that needs to be addressed in future experiments. Such interaction could modulate vascular formation since nestin-expressing cells play a role in angiogenesis during wound healing [51].

Based on the finding of CD34 colocalization with neuropeptide receptors, it is possible that sensory neurons are not only limited to modulate functional aspects of bulge SCs during wound healing, but also during hair growth cycle. Indeed, the capsaicin-treated rats showed less fur regrowth after shaving and SP induce anagen in mice skin when administered during telogen phase [3], [21]. SP has also been associated with premature induction of catagen in models of auditive stress while CGRP does not promote anagen neither in culture of skin explants nor in vivo experiments [21], [52]. The specific role of each neuropeptide and other nerve-derived signals on distinct epithelial populations, with its possible combinations, needs to be determined. In particular, it will be important to investigate whether CGRP is involved in maintaining SC quiescence or in promoting cell differentiation. The latter seems to be the case since it was found that CGRP raises the expression of β-catenin and c-myc mRNA in human epidermal stem cells [53]. The activation of Myc promotes histone acetylation leading to stem cell differentiation [54]. Whatever the specific role of CGRP, recent findings denote the relevance of CGRP signaling for nerve-epithelial interactions. It has been shown that keratinocytes increase the expression of the β isoform of CGRP in conditions of chronic pain, nerve damage, and inflammation [46]. This could mean that keratinocytes compensate subnormal availability of the α CGRP isoform (which is predominantly expressed in sensory neurons) when certain alterations of the nerve supply occur. This circumstance, however, may contribute to chronic pain mechanisms [46].

In summary, our work gives new insights into the efferent role that dorsal root ganglion neurons have on reepithelialization during wound healing in rodent skin. In addition, it highlights the importance of neural regulation on epithelial SC physiology as it was also described for the hematopoietic SCs which are modulated by sympathetic neurons [13]. Interestingly, sensory and sympathetic nerves coexist in several peripheral targets. This issue opens the possibility that both systems could act in concert to regulate diverse aspects of adult stem cells by a myriad of nerve-derived signals. The complexity of this scenario is beyond the communication via classical neurotransmitters and neuropeptides. Recently, it has been shown in elegant experiments that nonpeptidergic nerves use Hedghog signaling to maintain a subpopulation of follicular stem cells that are able to convert into epidermal progenitors [49]. In the future, it will be essential to understand the molecular pathways that the nervous system modulate to understand how neurons regulate the activation and differentiation of SC in different niches of the body and its possible implications for tumor formation.

## Materials and Methods

### Ethics Statement

In this study we used male Wistar rats raised in the animal facility of the Instituto de Investigaciones Biomédicas of the Universidad Nacional Autónoma de México. Rats were kept in a colony room at 21°C on a 12 h illumination cycle (lights on at 6∶00 am) with free access to food and water. The experimental procedures were approved by the Comisión para el Cuidado y Uso de Animales de Laboratorio (permit # 077) and carefully followed the guidelines of the *Lineamientos para el cuidado y uso de animales de laboratorio* published by the Instituto de Investigaciones Biomédicas (Código Ético IBB, 2005).

### Chemical Denervation

To reduce sensory innervation we used the neonatal capsaicin treatment as previously described [31]. Within 24–36 h after birth, hypothermia-anaesthetized rat pups were injected subcutaneously in the back skin with either a single dose of capsaicin (50 mg/kg) or vehicle alone (10% Tween-80 and 10% ethanol (v/v) in 0.9% NaCl). Once the rat pups had recovered from anaesthesia and capsaicin effect, they were returned to their mothers. Rat pups were weaned at 4 weeks.

### Wounding

Rats were anesthetized by inhalation of isoflurane (4% for induction, 1.5% maintenance) at eight weeks after capsaicin injection which corresponds to the telogen phase of the hair cycle growth in the rat [55]. The dorsal skin corresponding to thoracic vertebrae was shaved and cleaned with 70% ethanol. Then, we made a full-thickness wound with a dermal biopsy punch of 6 mm of diameter. Wounds were left uncovered and in the cases of bleeding it was cleaned with sterile cotton. At the end of the surgery, the rats were placed individually in a cage until they completely recover from anesthesia. Finally, the rats were returned to its original cage.

### Proliferation

To determine the effect of capsaicin denervation on the activation of epidermal and follicular progenitors, rats were injected with a single dose of bromodeoxyuridine (50 mg/kg) 21 h following wounding. Skin samples were collected at 31 h, 47 h, and 61 h postwounding. The rats (n = 5) were anesthetized with sodium pentobarbital and were transcardially perfused with 0.9% NaCl followed by Zamboni fixative [4% paraformaldehyde; 15% (v/v) saturate picric acid, 0.1 M phosphate buffer, pH 7.4]. The skin samples were postfixed for 24 h in the same fixative. Then, the samples were successively transferred to 20% and 30% sucrose. The tissue was embedded in OCT compound (Sakura Seiki Inc, Tokyo, Japan) and frozen in an orientation that allow to obtain longitudinal sections of hair follicles, as previously described [56].

### Migration

We used a double-labeling technique with two analogs of thymidine to evaluate whether sensory denervation alters the mobilization of transit amplifying cells from the follicle to the epidermis [7], [57]. This technique is based on the principle that follicular keratinocytes divide faster than the epidermal keratinocytes [7]. For this reason, rats (n = 5) were injected 21 h after wounding with a single dose of iododeoxyuridine (IdU; 150 nmol/g). Ten hours later (31 h postwounding) we injected a single dose of chlorodeoxyuridine (CldU; 150 nmol/g). Skin samples were collected at 32, 41, and 61 h postwounding and processed as described above. For all quantitative procedures, the skin sections were coded and the observers were blinded to the coding information.

### BrdU Immunohistochemistry

The samples were sectioned into 50 µm slices in a cryostat and the sections were individually collected in 48-well plates filled with cryoprotectant solution (25% ethylene glycol and 25% glycerol in a 0.05 M phosphate buffer) and stored at −20°C. For the analysis, sections were regionalized in two regions. The inner region comprised 2 mm from the edge of the wound, whereas the outer region spanned 2 mm further from the border of the inner region. Therefore, we collected 280 sections corresponding to a total region of 14 mm×14 mm. For stereological quantification, 14 sections were selected with a systematic random sampling. The sampling interval under this scheme was twenty, which implies that the first section was selected at random between the first and the twentieth section. Then, the series of sections were put onto gelatinized slides washed 2× with 0.1 M phosphate buffer (PB), rinsed 1× with 0.3% Triton X-100 in PB (PBT), and incubated with 1% H_2_O_2_ in PB for 1 h. After three PB rinses, sections were incubated with Immuno/DNA retriever at 70°C for 30 min (Bio SB, Santa Barbara, USA), and then washed with PB. For BrdU immunodetection, DNA was denatured with 1N HCl at 25°C for 30 min and neutralized with 0.1 M sodium borate buffer. After this step, the biotin-avidin blocking kit (Vector Laboratories, Burlingame, USA) was used to eliminate unspecific staining of the sebaceous gland of the hair follicle. The avidin (100 µL/mL) was diluted in PB and the sections were incubated for 30 min in this solution. After a rinse, the sections were incubated with a solution of biotin (100 µL/mL). Finally, sections were blocked with 5% normal horse serum in PBT for 1 h. The sections were incubated overnight with mouse anti-BrdU (1∶500, Roche Applied Science, Penzberg, Germany). After PB rinses, sections were incubated with biotinylated donkey anti-mouse (Chemicon, Temecula, USA) for 2 h and then with the avidin–biotin complex (Vector Laboratories) for 90 min. The immunohistochemistry reaction was made visible by 3-3′-diaminobenzidine/nickel precipitation (Vector Laboratories). After a rinse in water, sections were incubated in 0.05 M sodium bicarbonate buffer pH 9.6 for 10 min and then exposed to DAB enhancing solution (Vector laboratories). Finally, sections were counter-stained with methyl green.

### Quantification of Epithelial Proliferation

The total number of immunoreactive BrdU cells was estimated with the stereological probe of the optical fractionator [31]. Data were obtained with a Nikon Labophot-2 microscope equipped with 10× and 100× (oil immersion, 1.4 NA) objectives, a motorized x-y-z stage control, and interfaced with StereoInvestigator Software 9 (MBF Bioscience, Williston, USA). The boundaries of epidermis and follicle profiles were traced in the areas corresponding to the inner and outer regions described above. BrdU^+^ nuclei were counted at uniformly random sampled sites within the epidermal and follicular tracing. The size of the grid for the epidermis of the samples taken at 31 h postwounding was X = 927 µm and Y = 34 µm with a counting frame measuring 30×25 and a height of 21 µm.; for 47 h samples, X = 716 µm and Y = 78 µm with a counting frame measuring 30×25 and a height of 25 µm; and for 61 h samples, X = 480 µm and Y = 180 µm a counting frame measuring 30×25 and a height of 24 µm. The size of the grid for the follicular region of the samples obtained at 31 h was X = 172 µm and Y = 200 µm with a counting frame measuring 43×32 and a height of 22 µm; for 47 h and 61 h samples, X = 344 µm and Y = 180 µm with a counting frame measuring 43×32 and a height of 24 µm. The guard zone was 10% of the mean section thickness, which was measured at every sampling site.

### IdU/CldU Immunofluorescence

The skin samples labeled with IdU and CldU were also sectioned into 50 µm slices and preserved in cryoprotectant solution. By a systematic random sampling, we selected 10 sections with a sampling interval of twenty. This area corresponded to the inner region around the wound. The series of sections were washed 2× with Tris buffered saline (TBS, pH 7.4) and rinsed 1× with TBS containing 0.3% Triton X-100 (TTBS). Then, sections were incubated with Immuno/DNA retriever at 70°C for 30 min and then washed with TBS. Also sections were incubated with 1N HCl at 37°C for 1 h followed by a 10 min wash with 0.1 M sodium borate buffer. After three washes in TBS, sections were incubated 1 h in blocking solution containing 5% normal goat serum and 5% bovine serum albumin in TTBS. The primary antibodies were diluted in blocking solution and incubated 12 to 16 h at room temperature. The detection of the halogenated thymidne analogs was accomplished using mouse anti-BrdU (1∶1000; Becton Dickinson, clone B44) for IdU and rat anti-BrdU (1∶300; Serotec, clone BU1/75) for CldU [58]. After three washes, sections were incubated with donkey anti-mouse conjugated to Alexa 488 (1∶1,000) and donkey anti-rat conjugated to Alexa 584 (1∶1,500). After rinses in TBS, sections were counterstained with DAPI and coverslipped with Dako Fluorescence Mounting Medium (Dako, Carpinteria, USA).

### Quantification of Follicular Cell Migration

The estimation of the total number of IdU^+^/CldU^+^ in the epidermis and upper part of the hair follicle was obtained also by the optical fractionator probe. Data were obtained with an Olympus BX51 WI microscope equipped with a 4× and 60× (water immersion, 1.2 NA) objectives, a motorized x-y-z stage control, and the StereoInvestigator Software. The size of the grid for the epidermis of the samples obtained at 32 h postwounding was X = 392 µm and Y = 74 µm with a counting frame measuring 65×65 and a height of 30 µm; for 41 h and 61 h samples, X = 488 µm and Y = 82 µm with a counting frame measuring 65×65 and a height of 26 µm. The size of the grid for the follicular region in all the samples was X = 137 µm and Y = 250 µm. The counting frame measured 65×50 and a height of 30 µm (32 h) or 26 µm (41 h and 61 h). The guard zone in all the cases was 10% of the mean section thickness, which was measured at every sampling site. The percentage of the double labeled nuclei was obtained by determining the fraction of IdU^+^/CldU^+^ nuclei in relation to the total nuclei labeled with IdU.

### Evaluation of Programmed Cell Death

To qualitatively evaluate the presence of apoptotic cells in the epithelium of wounded skin we used the TUNEL assay and the immunolabeling for active caspase-3 on series of sections obtained by random sampling. TUNEL staining was performed with ApopTag Fluorescein Direct In Situ Apoptosis Detection Kit (Chemicon) according to the manufacturer’s instructions. Tissue permeabilization was achieved by incubating sections with proteinase K 20 µg/mL for 20 min at room temperature. For caspase-3 immunolabeling, sections were rinsed with PB and PBT and incubated with Immuno/DNA retriever. After an hour of blocking with 5% horse serum, sections were incubated overnight with rabbit polyclonal anti-caspase-3 (1∶500, Abcam, Cambridge, UK). After PB rinses sections were incubated with donkey anti-rabbit conjugated to Alexa 594. Finally, sections were counterstained with DAPI and coverslipped with Dako medium.

### Volume Estimation of the Expression of Keratin 6

The immunofluorescence to detect epidermal expression of Keratin 6 (K6) was performed in 14 sections selected by uniform random sampling. The sections were rinsed 2× in PB and 1× in PBT. After incubation with Immuno/DNA retriever, the sections were blocked for 30 min with 5% horse serum in PBT and incubated for 12–15 h with the monoclonal antibody mouse anti-K6 (1∶500; Biocare Medical, USA). After PB rinses, sections were incubated for 2 h at room temperature with donkey anti-mouse conjugated to Alexa 594 (1∶500). Finally, sections were incubated with DAPI and coverslipped with fluorescence mounting medium.

The volume of the epidermis immunoreactive to K6 was estimated by the stereological method of Cavalieri using the Olympus BX51 WI microscope interfaced with StereoInvestigator. This method is based on the principle that the volume of an object of irregular shape can be obtained by considering the area of the object profiles that appear in sections separated by a known distance. In order to obtain the volume estimation, a uniform array of points with a known area per point is placed over the image of the sections. Then, the number of points hitting the K6^+^ epidermis was counted on each selected sections. The distance between points was 120 µm.

Photographic documentation of BrdU, IdU/CldU or CK6 immunostaining was performed by creating a seamless montage. Using a motorized stage, we collected a series of images from skin sections with overlapping edges and used the function “Photmerge” from Phothoshop to align, stitch, and blend them. The resulting high-resolution images were cropped to assemble the corresponding figures.

### CGRP^+^ Fiber Length Estimation

The total length of CGRP^+^ fibers in the inner and outer region around the wound was quantified by using the space balls method for design-based stereology [31]. The immunohistochemistry was performed according to the protocol for BrdU staining, but the step of HCl incubation was omitted. Fiber detection was accomplished using rabbit anti-CGRP (1∶10,000, Peninsula Labs, San Diego, USA). Data was obtained in the Olympus BX51 WI microscope interfaced with StereoInvestigator. The traced region included the living strata of the epidermis and the dermal region that contained the upper part of the hair follicles up to the bulge region. For the quantification, we considered fibers in the epithelium and in the fibrous component of the dermis. The stereological parameters were as follows: sampling grid, 711×78 µm; radius of the sphere, 12 µm; and guard zones, 13% of section thickness. In additional experiments, double immunofluorescence was performed with mouse anti-neural III beta-tubulin (1∶500, Promega, Madison, USA).

### Immunofluorescence for Neuropeptide Receptors

At postnatal day four, a group of control and capsaicin-treated rats were injected twice daily with BrdU (50 mg/kg) during three days. After 8 to 10 weeks, these rats were transcardially perfused with Zamboni fixative. Samples from back skin were collected, postfixed for 24 h in the same fixative, and successively transferred to 20% and 30% sucrose. Samples were sectioned into 30 µm slices and stored in cryoprotectant solution. The sections were incubated for 30 min in Immuno/DNA retriever solutions, for 1 h at 25°C in HCl, and 10 min in sodium borate. After TBS rinses, the sections were incubated for 1 h with 5% horse serum and then incubated overnight with a mixture of primary antibodies. We used the following primary antibodies: rabbit anti-neurokinin receptor 1 (NK-1; 1∶300, Chemicon), rabbit anti-calcitonin like protein (CLR; 1∶1,000), mouse anti-BrdU (1∶300, Roche Applied Science), goat anti-CD34 (1∶100, Santa Cruz laboratories, Santa Cruz, USA), mouse anti-nestin (1∶300, Chemicon), and rabbit anti-receptor activity modifying protein 1 (RAMP-1; 1∶100, Santa Cruz Laboratories). All the secondary antibodies were raised in donkey and included anti-mouse conjugated to Alexa 488, anti-rabbit conjugated to Alexa 594, and anti-goat Alexa 647 (Fig. S6). The sections were counterstained with either propidium iodide (PI) or with TOTO-3 depending of the secondary antibody combination. Sections were imaged on a Zeiss LSM5 Pascal confocal microscope equipped with Ar/HeNe laser. The combinations that we analyzed for the presence of neuropeptide receptors in the bulge region included: NK-1/BrdU/TOTO, NK-1/CD34/PI, CLR/BrdU/TOTO, CLR/CD34/PI, CLR/BrdU/TOTO, BrdU/CD34/TOTO, Nestin/CD34/PI, NK-1/Nestin/CD34, CLR/Nes/CD34 y RAMP1/CD34/TOTO.

### Statistics

Data are expressed as mean ± SEM. Statistical analysis was performed using Origin Pro 8. Statistical differences between groups were determined by a two-tailed Student’s t-test. Multiple comparisons were performed by using one-way ANOVA test followed by a Tukey and Holm-Sidack post hoc test. Any difference with p<0.05 was considered statistically significant.

## Supporting Information

Figure S1 **The number of epidermal IdU^+^/CldU^+^ cells increased over time only in control rats.** The total number of immunoreactive nuclei for IdU and CldU in the skin epithelium was estimated in the inner region to the wound edge (inCtl and inCap). The graphs show the estimated number of double-labeled cells in the hair follicles (A) and the epidermis (B).
(EPS)

Figure S2 **Apoptotic cell death evaluation.** To evaluate possible events of programmed cell death in the epithelia of capsaicin-treated rats we performed immunostaining for active caspase-3 (A–C) and TUNEL assay (E and F). In general, neither treated rats (B) nor control rats (A) presented labeling for caspase-3 at the epidermis or hair follicles. The immunostaining for caspase-3 was mainly observed at the granulation tissue in both groups (D). As a physiological control of cell death we used rat mammary gland obtained at the fourth day after weaning (C). A similar pattern of labeling was observed in the mammary gland using the TUNNEL assay (E). Panel D shows a unique observation where epidermal cells were positive for TUNNEL assay in a treated rat. These labeled nuclei were adjacent to a mass of disorganized material where no living strata were observed. epi, epidermis; der, dermis; sg, sebaceous gland; b, bulge; hf, hair follicle. Scale bar = 50 µm.
(TIF)

Figure S3 **CGRP^+^ fibers in the epidermis and hair follicles of wounded skin.** The panels show photomicrographs of CGRP immunostaining of skin sections of control (A, B, E, F) and treated rats (C, D, G, H) at 32 h (A, B, C, D) and 61 h (E, F, G, H) postwounding. The CGRP^+^ fibers (arrowhead) diminished with time in the control group. Nevertheless, the number of peptidergic fibers associated with skin epithelium was severely reduced in capsaicin-treated rats, independently of postwounding time. epi, epidermis; der, dermis; sg, sebaceous gland; b, bulge; hf, hair follicle; hs, hair shaft. Scale bar = 100 µm.
(TIF)

Figure S4 **In capsaicin-treated rats, the SP^+^ fibers were not associated with skin epithelium.** The panels show photomicrographs of substance P (SP) immunostaining of skin sections of control (A, B, E, F) and treated rats (C, D, G, H) at 32 h (A, B, C, D) and 61 h (E, F, G, H) postwounding. In control skin, the SP^+^ fibers (arrowheads) were located at the epidermis, at the subepidermal plexus, in the nearby of hair follicles, and in close association of blood vessels. In contrast, SP^+^ fibers in treated rats were only observed at blood vessels and in the deep dermis. epi, epidermis; der, dermis; sg, sebaceous gland; b, bulge; hf, hair follicle. Scale bar = 100 µm.
(TIF)

Figure S5 **Label-retaining cells but not nestin immunoreactive cells colocalize with neuropeptide receptors.** By confocal microscopy we evaluated the presence of SP receptor (NK-1, A) and CGRP receptor (CLR, B) on BrdU-retaining cells. The immunoreactivity for neuropeptide receptors was not observed in nestin-positive cells. These cells had a dendritic morphology and were located outside the outer root sheath of the hair follicle. Scale Bar = 20 µm.
(TIF)

Figure S6 **Neuropeptide receptors in the rat spinal cord.** The staining pattern for NK-1 (A and B), CLR (C and D), and RAMP-1 (E) was verified on spinal cord sections. The label for the three antibodies was mainly observed at the dorsal horn of the spinal cord as previously described. Panel F shows the RAMP-1 staining at the epidermis (F). Note that RAMP-1 immunoreactivity is localized in the basal layer of the epidermis. As background control, skin sections were incubated with only secondary antibodies. Panel G: anti-mouse conjugated to Alexa 488, anti-rabbit conjugated to Alexa 594, and anti-goat conjugated to Alexa 647; panel H: anti-mouse (Alexa 488), anti-rabbit (Alexa 594), and TOTO-3 counterstaining. The hair shaft presented autofluorescence with the three excitation laser lines used. The sebaceous gland showed faint staining with anti-rabbit antibody. epi, epidermis; der, dermis; sg, sebaceous gland; b, bulge; hf, hair follicle; hs, hair shaft; dh, dorsal horn. Scale bar: A and C = 100 µm; B, D, E, F, G, and H = 50 µm.
(TIF)
