# Supplementary figures and images for: Modulatory Role of Sensory Innervation on Hair Follicle Stem Cell Progeny during Wound Healing of the Rat Skin
Source: PLoS One. 2012 May 4;7(5):e36421. doi: 10.1371/journal.pone.0036421 (PMC3344885; doi:10.1371/journal.pone.0036421)

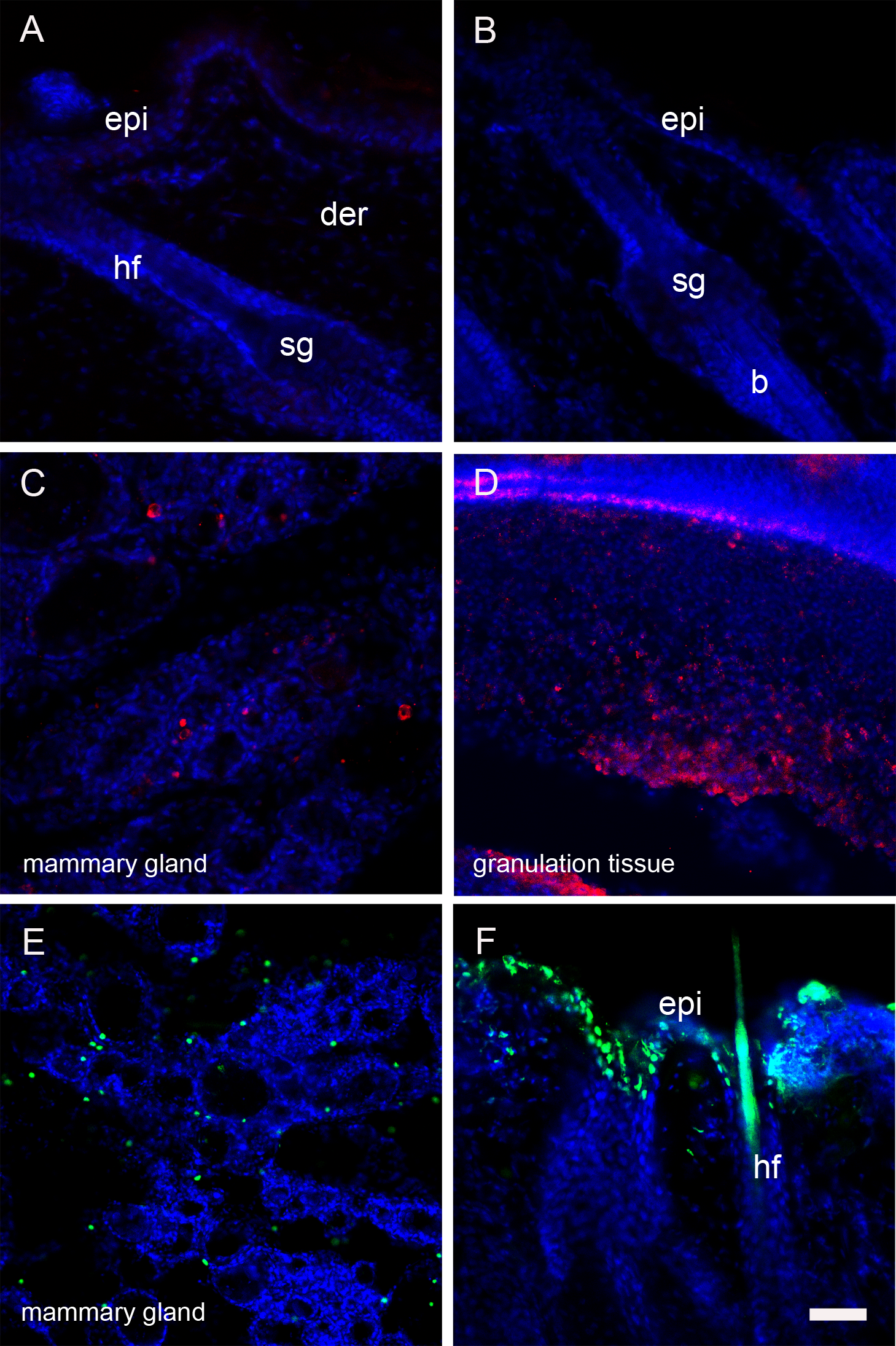

Supplement: Figure S2 — Apoptotic cell death evaluation. To evaluate possible events of programmed cell death in the epithelia of capsaicin-treated rats we performed immunostaining for active caspase-3 (A–C) and TUNEL assay (E and F). In general, neither treated rats (B) nor control rats (A) presented labeling for caspase-3 at the epidermis or hair follicles. The immunostaining for caspase-3 was mainly observed at the granulation tissue in both groups (D). As a physiological control of cell death we used rat mammary gland obtained at the fourth day after weaning (C). A similar pattern of labeling was observed in the mammary gland using the TUNNEL assay (E). Panel D shows a unique observation where epidermal cells were positive for TUNNEL assay in a treated rat. These labeled nuclei were adjacent to a mass of disorganized material where no living strata were observed. epi, epidermis; der, dermis; sg, sebaceous gland; b, bulge; hf, hair follicle. Scale bar = 50 µm. (TIF) [file pone.0036421.s002.tif]

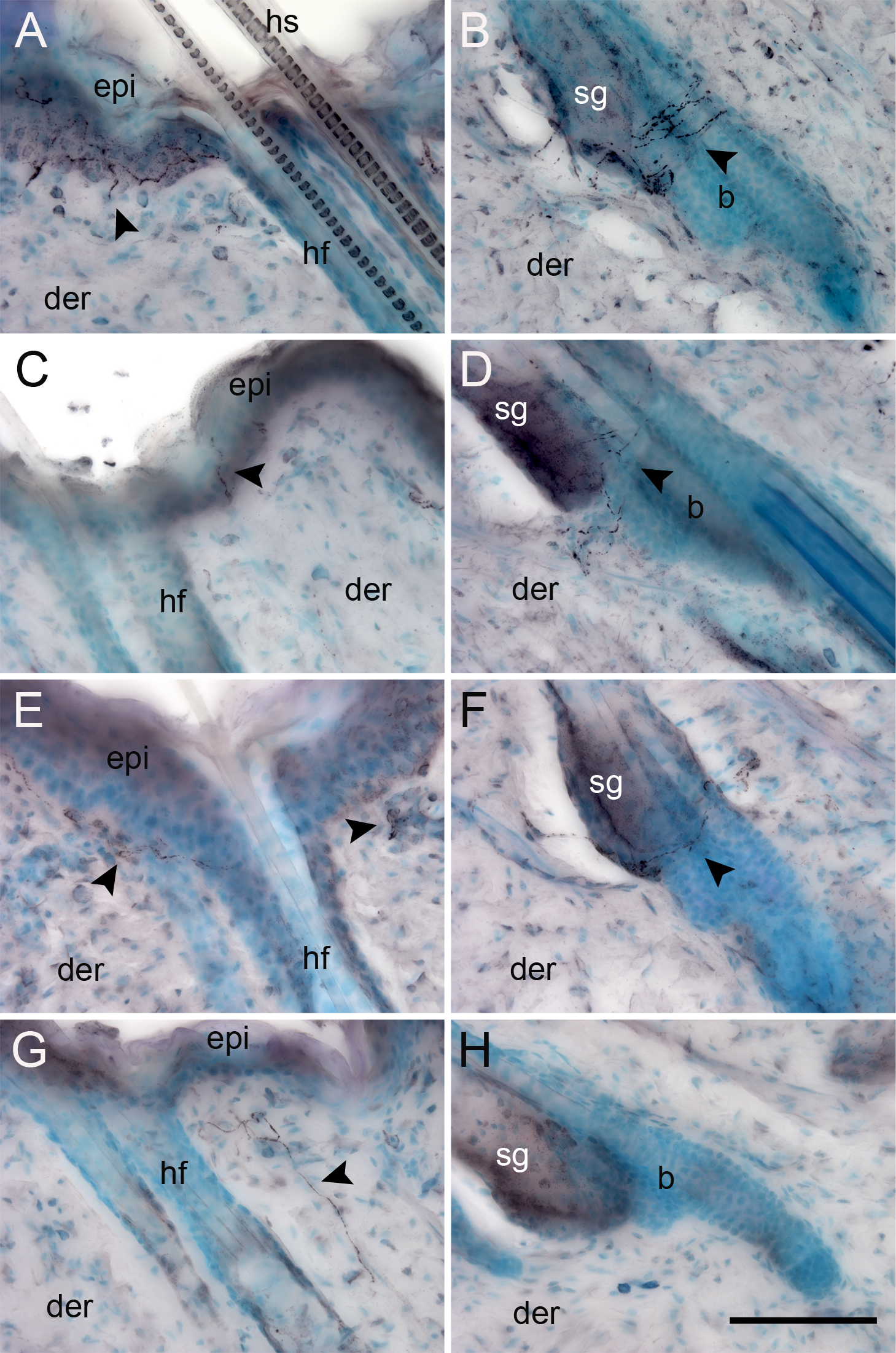

Supplement: Figure S3 — CGRP+ fibers in the epidermis and hair follicles of wounded skin. The panels show photomicrographs of CGRP immunostaining of skin sections of control (A, B, E, F) and treated rats (C, D, G, H) at 32 h (A, B, C, D) and 61 h (E, F, G, H) postwounding. The CGRP+ fibers (arrowhead) diminished with time in the control group. Nevertheless, the number of peptidergic fibers associated with skin epithelium was severely reduced in capsaicin-treated rats, independently of postwounding time. epi, epidermis; der, dermis; sg, sebaceous gland; b, bulge; hf, hair follicle; hs, hair shaft. Scale bar = 100 µm. (TIF) [file pone.0036421.s003.tif]

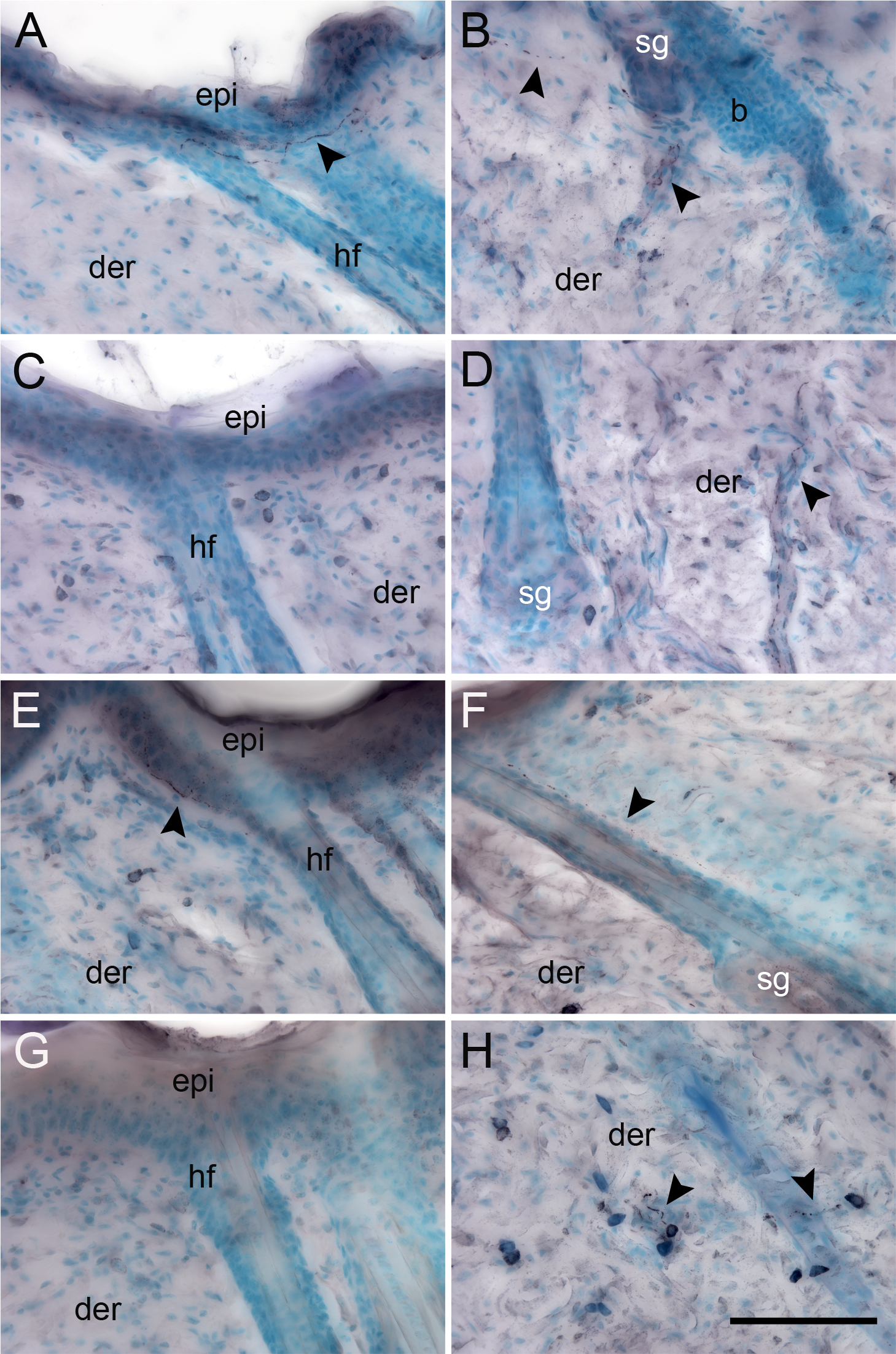

Supplement: Figure S4 — In capsaicin-treated rats, the SP+ fibers were not associated with skin epithelium. The panels show photomicrographs of substance P (SP) immunostaining of skin sections of control (A, B, E, F) and treated rats (C, D, G, H) at 32 h (A, B, C, D) and 61 h (E, F, G, H) postwounding. In control skin, the SP+ fibers (arrowheads) were located at the epidermis, at the subepidermal plexus, in the nearby of hair follicles, and in close association of blood vessels. In contrast, SP+ fibers in treated rats were only observed at blood vessels and in the deep dermis. epi, epidermis; der, dermis; sg, sebaceous gland; b, bulge; hf, hair follicle. Scale bar = 100 µm. (TIF) [file pone.0036421.s004.tif]

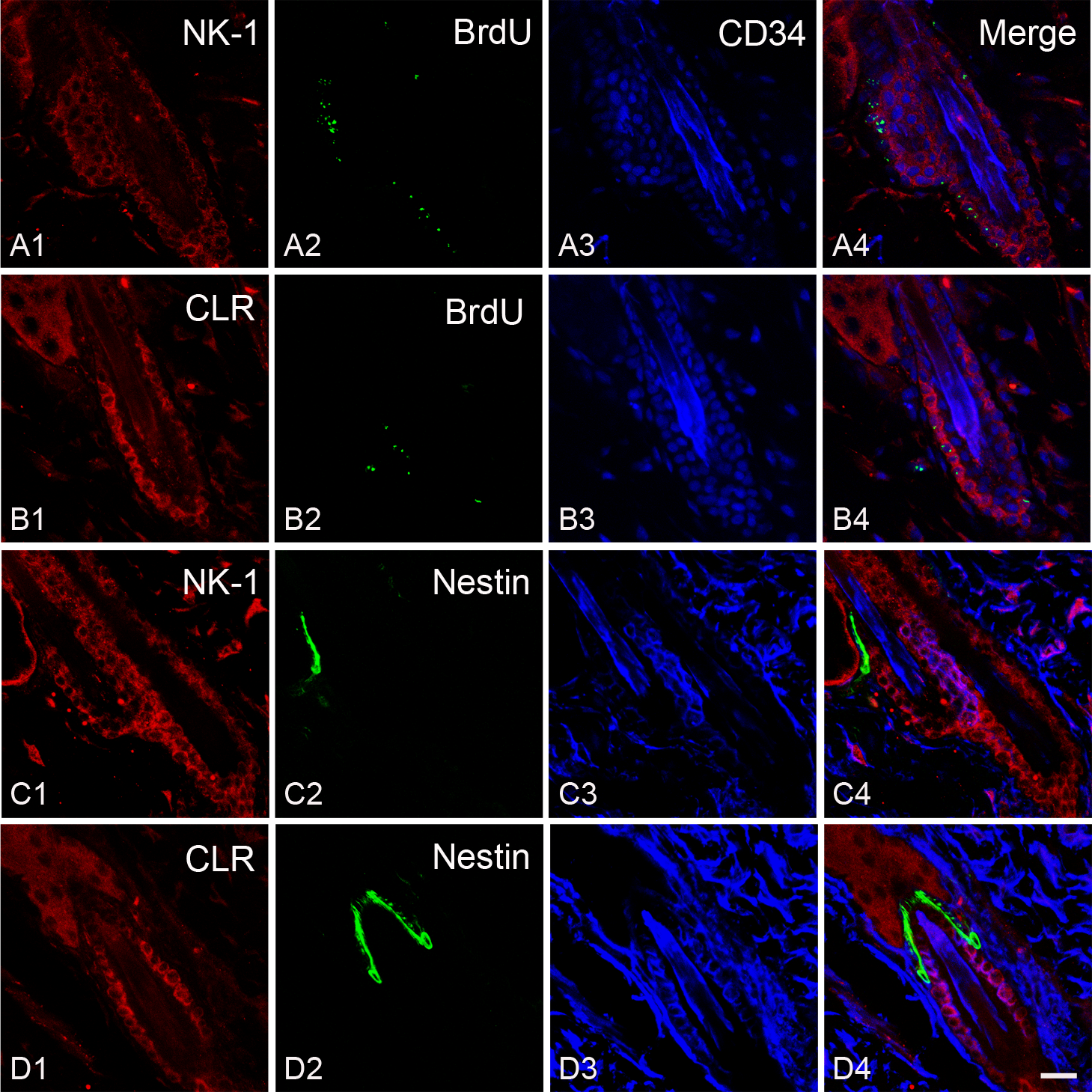

Supplement: Figure S5 — Label-retaining cells but not nestin immunoreactive cells colocalize with neuropeptide receptors. By confocal microscopy we evaluated the presence of SP receptor (NK-1, A) and CGRP receptor (CLR, B) on BrdU-retaining cells. The immunoreactivity for neuropeptide receptors was not observed in nestin-positive cells. These cells had a dendritic morphology and were located outside the outer root sheath of the hair follicle. Scale Bar = 20 µm. (TIF) [file pone.0036421.s005.tif]

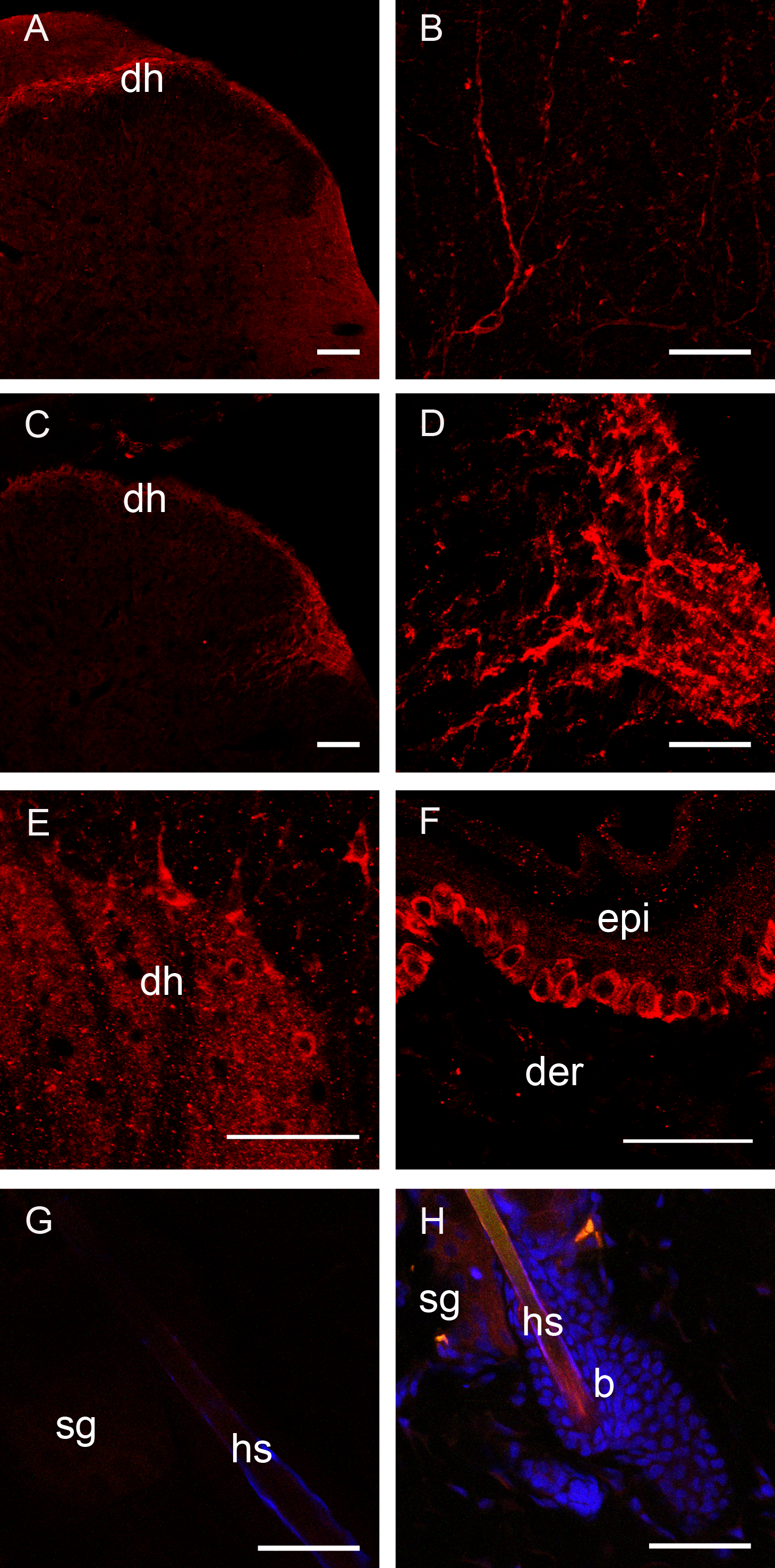

Supplement: Figure S6 — Neuropeptide receptors in the rat spinal cord. The staining pattern for NK-1 (A and B), CLR (C and D), and RAMP-1 (E) was verified on spinal cord sections. The label for the three antibodies was mainly observed at the dorsal horn of the spinal cord as previously described. Panel F shows the RAMP-1 staining at the epidermis (F). Note that RAMP-1 immunoreactivity is localized in the basal layer of the epidermis. As background control, skin sections were incubated with only secondary antibodies. Panel G: anti-mouse conjugated to Alexa 488, anti-rabbit conjugated to Alexa 594, and anti-goat conjugated to Alexa 647; panel H: anti-mouse (Alexa 488), anti-rabbit (Alexa 594), and TOTO-3 counterstaining. The hair shaft presented autofluorescence with the three excitation laser lines used. The sebaceous gland showed faint staining with anti-rabbit antibody. epi, epidermis; der, dermis; sg, sebaceous gland; b, bulge; hf, hair follicle; hs, hair shaft; dh, dorsal horn. Scale bar: A and C = 100 µm; B, D, E, F, G, and H = 50 µm. (TIF) [file pone.0036421.s006.tif]
